# Preventing dentin erosion with silver diamine fluoride and salivary pellicle: an in vitro study

**DOI:** 10.1007/s00784-024-05988-6

**Published:** 2024-10-08

**Authors:** Darren Dhananthat Chawhuaveang, Walter Yu-Hang Lam, Chun-Hung Chu, Ollie Yiru Yu

**Affiliations:** Faculty of Dentistry, The University of Hong Kong, 3B12, Prince Philip Dental Hospital, 34 Hospital Road, Hong Kong SAR, 999077 China

**Keywords:** Dental erosion, Dentin, Fluoride, Silver diamine fluoride, Salivary pellicle, Remineralization

## Abstract

**Objectives:**

To investigate the preventive and discoloring effects of a single and two weekly applications of 38% silver diamine fluoride (SDF) against dentin erosion.

**Materials and methods:**

180 dentin blocks were divided into four groups. Group 1 (SDF2) received two weekly applications of 38% SDF. Group 2 (SDF1) received a single application of 38% SDF. Group 3 (SNF) received a daily application of stannous chloride/amine fluoride/sodium fluoride (standard of care for dental erosion). Group 4 (DW) received a daily application of deionized water. The treated blocks were subjected to a 14-day erosive challenge. Crystal characteristics, elemental composition, surface morphology, percentage of surface microhardness loss (%SMHL), surface loss, and color change (ΔE) were investigated using X-ray diffraction (XRD), energy-dispersive spectrometry (EDS), scanning electron microscopy (SEM), hardness testing, profilometry, and digital spectrophotometry, respectively.

**Results:**

XRD and EDS showed dentin surfaces had silver compounds in SDF2 and SDF1, and stannous chloride in SNF. SEM revealed less dentin demineralization with tubular occlusion in SDF2, SDF1, and SNF, but severe demineralization in DW. The %SMHL of SDF2, SDF1, SNF, and DW were 10.8 ± 2.1, 15.7 ± 2.1, 17.9 ± 2.1, and 28.7 ± 2.0 (SDF2 < SDF1 < SNF < DW, *p* < 0.05). Surface loss (µm) of SDF2, SDF1, SNF, and DW were 5.0 ± 0.6, 6.0 ± 0.6, 6.0 ± 0.7, and 9.0 ± 0.5 (SDF2 < SDF1 = SNF < DW, *p* < 0.001). ΔE of SDF2, SDF1, SNF, and DW were 26.0 ± 3.4, 12.1 ± 3.8, 6.9 ± 3.5, and 3.9 ± 3.6 (SDF2 > SDF1 > SNF = DW, *p* < 0.001).

**Conclusion:**

38% SDF with two weekly applications provided better preventive effects against dentin erosion, but it might discolor dentin.

**Clinical relevance:**

The increased 38% SDF application showed a better anti-erosive potential against dentin erosion. However, SDF caused black staining on the dentin.

## Introduction

Dental erosion is mineralized tooth substance loss due to chemical acids not generated by oral microorganisms [1, 2]. Erosive acids can be classified into two categories: endogenous acids derived from the reflux of gastrointestinal acids and exogenous acids mainly derived from diet, medications, or occupational acidic exposure [1, 3]. The global prevalence of dental erosion has increased sharply over the past decades [4–7]. Initial erosive lesions on the enamel surface can progress to the dentin surface, indicating advanced erosive lesions [1]. In addition, root surface exposure caused by gingival recession, particularly in the older adult population, increases the risk for acidic demineralization on root dentin surfaces, thereby increasing the risk of dentin erosion [3]. Unfortunately, most patients with dental erosion remain symptom-free and lack awareness until the disease reaches an advanced stage [8, 9]. If dental erosion progresses to the dentin surface, it can be associated with dentin hypersensitivity, pain, esthetic problems, and even tooth fracture [2, 6, 10]. Preventing and managing dental erosion require to control the risk factors of the disease [1–3]. Moreover, topical anti-erosive agents have been recommended for oral health care to protect teeth from dental erosion [10–12].

The most common topical anti-erosive agent is fluoride [10]. Many commercial fluoride products, such as amine fluoride (AmF), sodium fluoride (NaF), stannous fluoride (SnF_2_), and stannous chloride-containing fluoride solution (SnCl_2_/AmF/NaF), have been applied for dental erosion prevention [13, 14]. Currently, the combination of SnCl_2_/AmF/NaF solution is the standard care for dental erosion prevention [10, 13, 14]. These fluoride agents could strengthen the tooth surface and prevent dental erosion by modifying hydroxyapatite and creating a precipitated layer after application [10, 15, 16]. However, their anti-erosive effects were inconclusive [10, 17]. Furthermore, it requires daily application or a high concentration of fluoride in the formula to reach the level of protection against dental erosion [10, 18].

Silver diamine fluoride (SDF) is an alkali solution containing a high concentration of fluoride and silver ions [19, 20]. SDF has been widely used for caries management. However, its effectiveness in managing dental erosion, especially on dentin surface, needs further investigation. Our previous in vitro study reported that a single application of 38% SDF led to lower surface loss and lower percentage surface microhardness loss of human dentin surfaces compared to the control [9]. Likewise, Ainoosah et al. found that 38% SDF reduced surface loss in bovine dentin [21]. The possible anti-erosive properties of SDF on dental erosion could be due to the reaction of SDF with the teeth’s minerals to form a precipitated layer of silver compounds on the surface [9, 19]. It can modify hydroxyapatite crystal to enhance it more acid-resistant [9, 10]. It also inhibits matrix metalloproteinases (MMPs) to preserve demineralized organic matrix (DOM), thereby delaying acid diffusion and reducing dentin erosion progression [10, 22].

The frequency of SDF application is an essential factor contributing to its anti-erosive effect [10, 23]. The common frequency of SDF application for dental caries management is either every six months or once a year [24]. However, no studies have investigated the frequency of SDF application in preventing dentin erosion. Besides, there is no consensus on the SDF application protocol for anti-erosion purposes on dentin surfaces. Hence, the anti-erosive effect of SDF with different application protocol on dentin needs to be investigated. In addition, SDF applications may cause a permanent black stain on human eroded dentin with exposed collagen [19]. Therefore, this study aimed to investigate the preventive and discoloring effects of 38% silver diamine fluoride solution with two weekly topical applications or a single against dentin erosion. The null hypothesis was that the applications frequency would not affect the preventive and discoloring effect of 38% SDF solution against dentin erosion compared to control groups.

## Materials and methods

The Institutional Review Board of the University of Hong Kong/Hospital Authority Hong Kong West Cluster approved this study (IRB: UW 20–210).

### Preparation of dentin samples

One hundred eighty extracted human third molars from patients aged 18–30 were collected under an IRB-approved protocol. All extracted third molars were cleaned and stored in 0.1% thymol solution at 4 °C before use. A dentin block with a size of 4 mm width × 4 mm length × 2 mm thickness was sectioned from the mid-coronal part of the crown by a low-speed cutting machine (ISOMET 1000, Buehler, Lakebluff, Illinois, USA). Each dentin block was polished with waterproof silicon carbide paper (1000, 1200, and 4000 grit) under running water. The polished dentin block was examined by 10X magnification of a stereomicroscope to ensure no cracks or caries were present. The polished dentin blocks were cleaned in an ultrasonic bath for 5 min to remove polishing debris. Two layers of acid-resistant nail polish covered on the left and right sides of dentin blocks with an exposed 2 mm width × 4 mm length window to standardize the experimental area.

### Experimental treatments

**Fig. 1:**
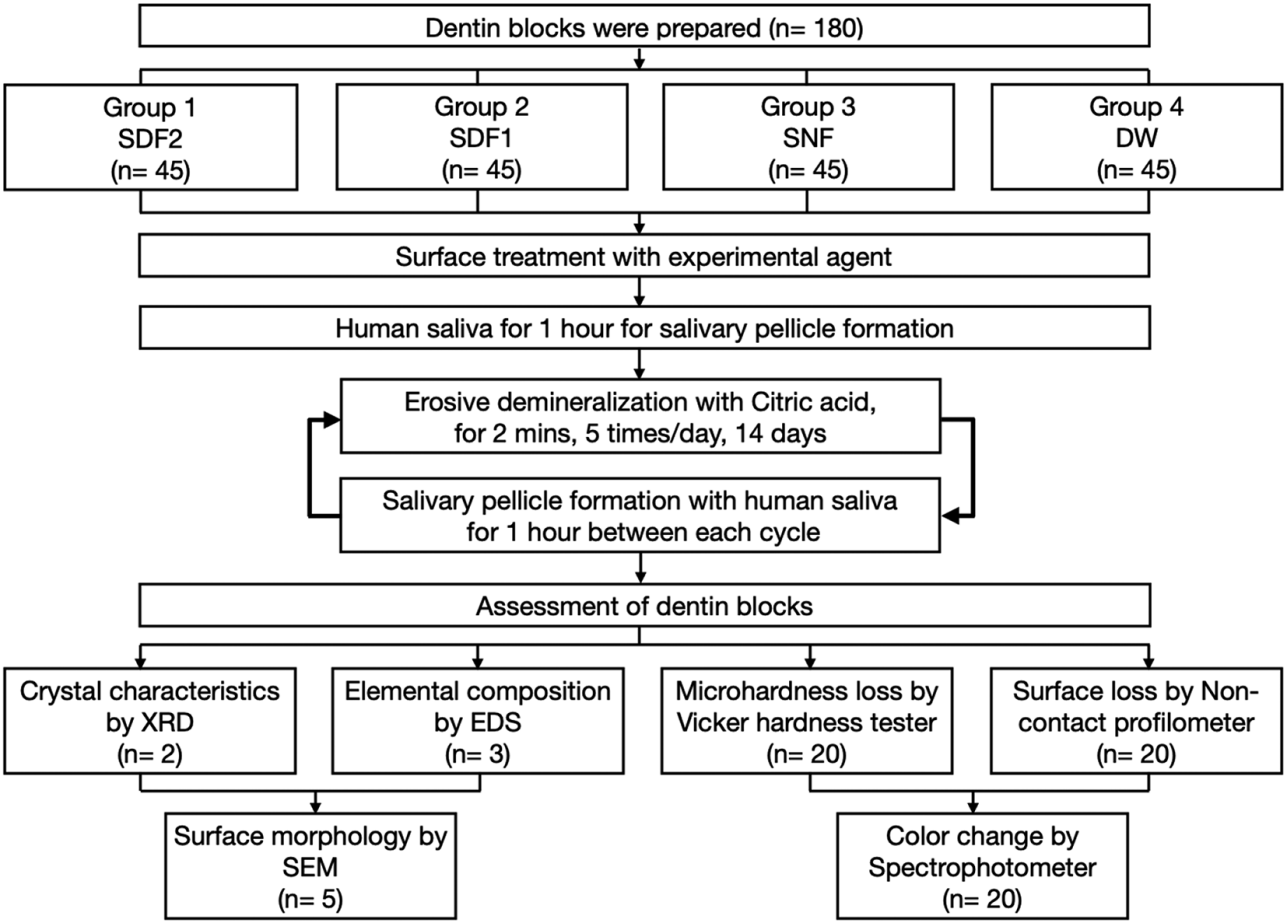
Flowchart of the experimental procedures. SDF: silver diamine fluoride; SNF: stannous chloride-containing fluoride solution; DW: deionized water; min: minute; XRD: X-ray diffraction; EDS: Energy-dispersive X-ray spectroscopy; SEM: scanning electron microscopy

The flowchart of the experimental procedures in this study is shown in Fig. 1. All experimental agents in this study were commercially available products in Hong Kong SAR distributors. 38% silver diamine fluoride (SDF contains 260,000 ppm Ag^+^ and 44,300 ppm F^−^, pH 10, Advantage arrest, Elevate Oral Care, West Palm Beach, FL, USA). One drop of 38% SDF solution is approximately 25 µl, which can treat five surfaces [25], and stannous chloride-containing fluoride solution (SNF contains 800 ppm Sn^2+^of SnCl_2_ and 500 ppm F^−^ of AmF/NaF; SnCl_2_/AmF/NaF, pH 4.5, Elmex Enamel Professional tooth rinse, GABA Schweiz, Colgate-Palmolive, Therwil, Switzerland). One hundred eighty prepared dentin blocks were divided into the following four groups (*n* = 45 for each group) according to sample randomization by a research randomizer program.

Group SDF2, 5 µl of 38% SDF was pipetted onto the dentin block and applied by a micro-brush onto the surface twice, once on day 1 and once on day 8 of the experiment. The dentin block was then air-dried for 1 min.

Group SDF1, 5 µl of 38% SDF was pipetted onto the dentin block and applied by a micro-brush onto the surface in a single application on day 1 of the experiment. The dentin block was then air-dried for 1 min.

Group SNF, Each dentin block was immersed in 1 mL of SNF solution for 30 s daily for 14 consecutive days. The dentin block was then air-dried for 1 min.

Group DW, Each dentin block was immersed in 1 mL of DW for 30 s daily for 14 consecutive days. The dentin block was then air-dried for 1 min.

### Collection of whole mouth stimulated saliva

Ten saliva donors (five women and five men) with a mean age of 32 ± 4 years were recruited. The inclusion criteria of saliva donors were nonsmokers and nonpregnant women. Saliva donors presented good oral health and the saliva donors did not present active caries or distinct defects of hard tissue loss of more than 50% of the surface area due to erosive tooth wear or periodontal disease. Moreover, their stimulated salivary flow rate was > 1 mL/min and unstimulated salivary flow rate was > 0.25 mL/min. The saliva collection was performed between 8 and 9 am from five saliva donors and 1–2 pm from another five saliva donors. The saliva donors were instructed to abstain from food and drink for one hour before saliva collection. To stimulate saliva secretion, the donor chewed a piece of paraffin film (2 cm × 2 cm) and spat the stimulated saliva into a 50 mL tube for 15 min. The saliva of all saliva donors was pooled and salivary pH was measured. The salivary pH was measured by a pH Meter (Hanna HI 2211 pH/ORP meter, Kehl, Germany). The mean salivary pH in the 14-day experiment was 6.98 ± 0.10.

### Erosive challenge

After the experimental treatment, each dentin block was immersed in 0.5 mL fresh human saliva under 70 rpm at 37 °C for one hour to allow the formation of a salivary pellicle [9, 26]. For the erosive challenge, each dentin block was immersed in 2 mL of 0.3% citric acid solution at pH 3.2 for 2 min with agitation at 70 rpm at 37 °C [27, 28]. The erosive challenge was performed 5 times per day at one-hour interval for 14 consecutive days. The dentin blocks were removed from the acid solution, washed with DW for 30 s, and gently dried with a soft absorbent paper. During each one-hour interval of erosive challenge and at the end of each day, each dentin block was immersed in 0.5 mL stimulated human saliva with agitation at 70 rpm at 37 °C. The citric acid and stimulated human saliva were renewed every cycle.

### Crystal characteristics measurement

The crystal precipitated and crystal structure of the dentin surface were analyzed using X-ray diffraction (XRD, Bruker AXS Model D8 Advance, Karlsruhe, Germany) (*n* = 2 for each group). The XRD data were collected with an X-ray diffractometer with CuKa (l = 1.5406 A◦) radiation. The parameters were set at 20–60° 2q, voltage = 40 kV, current = 40 mA, increment = 0.02 degree/step, and scan speed = 0.6 s/step. The phase purity and indexing of the chemical phase were checked according to the database of the International Centre for Diffraction Data (ICDD, PDF-4+/Web 2022) [29].

### Elemental composition measurement

The element composition of the precipitated on the dentin surface was analyzed using scanning electron microscopy (SEM, Hitachi S-4800 FEG Scanning Electron Microscope, Hitachi Ltd., Tokyo, Japan) under energy-dispersive X-ray spectroscopy (EDS) analysis (*n* = 3 for each group). The mean weight% (wt%) of calcium (Ca), phosphorus (P), fluorine (F), chlorine (Cl), silver (Ag), and stannous (Sn) were measured at a 500X magnification, 5 kV of accelerating voltage, and 50 mA of beam current.

### Surface morphology measurement

The dentin surface morphology was observed using scanning electron microscopy (SEM, Hitachi S-4800 FEG Scanning Electron Microscope, Hitachi Ltd., Tokyo, Japan) at a magnification of 5,000X and 5 kV in a high-vacuum mode (*n* = 5 for each group; 2 dentin blocks from crystal characteristics and 3 dentin blocks from elemental composition measurement). Dentin blocks were fixed in 2.5% glutaraldehyde at 4 °C overnight. The dentin blocks were then dehydrated in an ascending series of alcohol (70%, 85%, and 95% for 10 min and 100% for 20 min). The dentin blocks were dehydrated in a desiccator (Leica EM CPD300, Leica Microsystems, Germany) and sputter-coated with gold/palladium (Quorum 150T ES plus, Quorum, United Kingdom).

### Surface microhardness loss measurement

The microhardness value of the dentin blocks was measured using a Vickers microhardness tester (FM-800, Future-Tech Corp, Japan) (*n* = 20 for each group) [30–32]. Dentin blocks were kept moistened to avoid dentin organic matrix shrinkage [33]. The dentin blocks were then carefully dried with a soft absorbent paper before assessing the microhardness value. The dentin block was loaded with a pressure of a 300 g load and 15 s dwell time under 10X magnification at the reference area before the experiment and at the treated area after a 14-day erosive challenge [34, 35]. Four indentations at intervals of 100 μm were made at the center of the treated and reference areas of each block, respectively. The mean value of the Vicker hardness number (VHN) of treated and reference areas was determined by averaging the VHN value of the four indentations. The mean VHN values were then used to calculate the percentage surface microhardness loss (%SMHL) using the following formula: %SMHL = {(VHN_ref_ – VHN_treated_) / VHN_ref_} $\:\times\:$ 100 [36, 37], where VHN_ref_ is the VHN at the reference area and VHN_treated_ is the VHN at the treated area.

### Surface loss measurement

The surface loss of the dentin blocks was measured using a non-contact profilometer (Infinite Focus SL, Alicona, Austria) (*n* = 20 for each group). The nail varnish was removed by acetone to expose the left and right sides of the reference area. The dentin blocks were stored in DW before the measurement. All measurements of dentin surface loss were made under constant moisture control [33]. The dentin block was positioned parallel to the horizontal plane. The surface scan was performed with a 20 mm objective, a vertical resolution of 150 nm, and a lateral resolution of 10 μm [33, 38, 39]. Two surface areas of each dentin block was scanned and captured. Each captured area included the left side of the reference area, the treated area, and the right side of the reference area. The captured areas were analyzed with Alicona MeasureSuite software (Version 5.1, Alicona Imaging GmbH, Alicona, Austria). The dentin surface loss (µm) was determined by averaging both values of the captured areas.

### Color change measurement

The color change of the dentin surfaces was measured using A VITA Easyshade Advance Portable Dental Spectrophotometer (VITA Zahnfabrik GmbH, Bad Säckingen, Germany) (*n* = 20 for each group; 10 dentin blocks from surface microhardness loss measurement and 10 dentin blocks from surface loss measurement). Color change of the dentin blocks were measured and recorded before the experiment treatment and after the 14-day erosive challenge. The spectrophotometer was calibrated before each examination according to the manufacturer’s instructions. Each dentin block was dried and assessed with the spectrophotometer. The Commission International del’Eclairage (CIE) L* a* b* color system is defined as the color on the dentin surface within a three-dimensional color space. The L* axis represented lightness ranging from black (0) to white (100), the a* axis represented red (+ a*) to green (-a*), and the b* axis represented yellow (+ b*) to blue (-b*). The total color change (ΔE) of the dentin blocks was calculated according to the formula of ΔE = {(L_before_*-L_after_*)^2^ + (a_before_*-a_after_*)^2^ + (b_before_*-b_after_*)^2^}^1/2^ [40–42].

### Statistical analysis

The sample size was determined based on our data of surface loss in a previous study [9]. The calculation was performed using G*Power software version 3.1 (Kiel University, Germany) with an effect size of 0.55, $\:\alpha\:$ = 0.05, power 1-$\:\beta\:$ = 0.8. The computed total sample size was 11 blocks per group for surface loss measurement. Then, 20 dentin blocks per group were used in surface loss measurement to ensure statistical power. The percentage surface microhardness loss used the same number of dentin blocks as the surface loss measurement. All data were analyzed using statistical analysis software power (SPSS version 29.0, IBM, Armonk, New York, United States). Normality was tested with the Kolmogorov-Smirnov test. The homogeneity of variance was checked using the Levene test. One-way ANOVA and Tukey’s multiple comparison tests were used to detect the differences in percentage surface microhardness loss, surface loss, and total color change values among groups. The significant level for statistical analysis was set at 0.05.

## Results

### Crystal characteristics

**Fig. 2:**
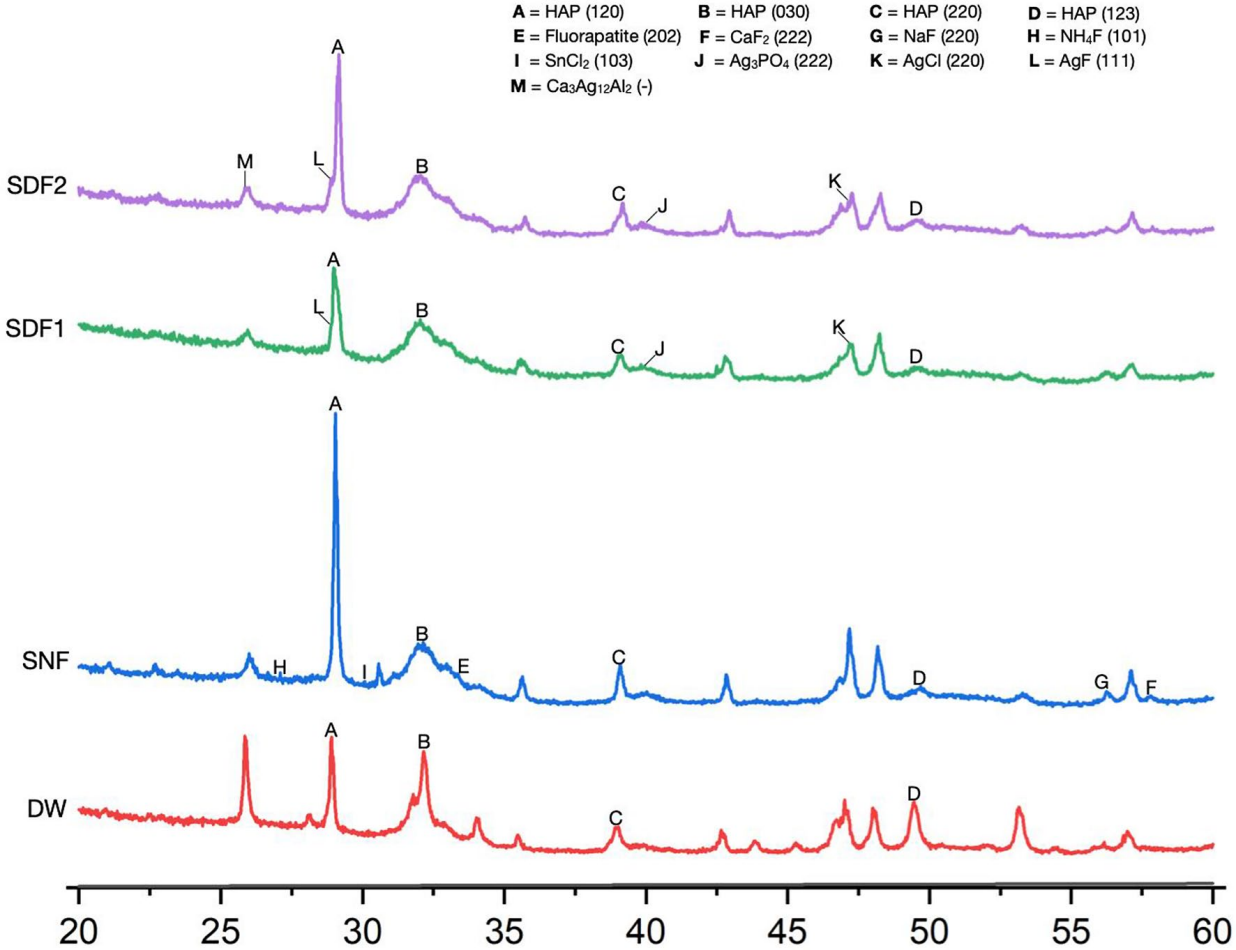
Typical X-ray diffraction patterns of the dentin in the four treatment groups. SDF: silver diamine fluoride; SNF: stannous chloride-containing fluoride; DW: deionized water; HAP: Hydroxyapatite; CaF_2_: Calcium fluoride; NaF: Sodium fluoride; NH_4_F: Ammonium fluoride; SnCl_2_: Stannous chloride; Ag_3_PO_4_: Silver phosphate; AgCl: Silver chloride; AgF: Silver fluoride; Ca_3_Ag_12_Al_2_: Calcium silver aluminum

Figure 2 shows the typical XRD spectra of the dentin blocks. The diffraction peaks of hydroxyapatite (HAP) were detected at 28.930°, 32.906°, 38.179°, and 49.490° in all the groups, consistent with (120), (030), (220), and (123) Bragg reflections of the hexagonal crystal structure of HAP. In both Groups SDF2 and SDF1, the peak at 28.917° coincided with silver fluoride (AgF, 111), the peak at 40.471° coincided with silver phosphate (Ag_3_PO_4_, 222), and the peak at 47.026° coincided with silver chloride (AgCl, 220) Bragg reflections, indicating the formation of the cubic crystal structure of AgCl and Ag_3_PO_4_ and the orthorhombic crystal structure of AgF on the dentin blocks. The peak at 25.927° coincided with calcium silver aluminum (Ca_3_Ag_12_Al_2_, 002) were detected in Group SDF2 only, representing the formation of the cubic crystal structure of Ca_3_Ag_12_Al_2_. In Group SNF, the peak at 26.261° coincided with ammonium fluoride (NH_4_F, 101), the peak at 34.141° coincided with fluorapatite (202), the peak at 56.099° coincided with sodium fluoride (220), the peak at 58.475° coincided with calcium fluoride (CaF_2_, 222), and the peak at 31.294° coincided with stannous chloride (SnCl_2_, 103), Bragg reflections, representing the formation of the hexagonal crystal structure of NH_4_F and fluorapatite, the cubic crystal structure of CaF_2_ and NaF and the orthorhombic crystal structure of SnCl_2_ on the dentin blocks. Table 1 shows the phase identification analysis of other crystal compounds formed on the dentin block in each treatment group.

**Table 1:** Phase identification was analyzed by the International Centre for Diffraction Data, Powder Diffraction file™, in the four experimental groups of dentin samples

Compound name (Crystal structure)	Experimental groups			
	SDF2 Conc. level	SDF1 Conc. level	SNF Conc. level	DW Conc. level
Hydroxyapatite-HAP (Hexagonal)	25.7%	30.8%	10.1%	100%
Fluorapatite-Ca_5_(PO_4_)_3_F (Hexagonal)	**×**	**×**	24.1%	**×**
Calcium fluoride-CaF_2_ (Cubic)	**×**	**×**	19.6%	**×**
Sodium fluoride-NaF (Cubic)	**×**	**×**	16.5%	**×**
Ammonium Fluoride-NH_4_F (Hexagonal)	**×**	**×**	3.8%	**×**
Stannous chloride-SnCl_2_ (Orthorhombic)	**×**	**×**	25.9%	**×**
Silver phosphate-Ag_3_PO_4_ (Cubic)	49.0%	58.2%	**×**	**×**
Silver chloride-AgCl (Cubic)	5.6%	7.3%	**×**	**×**
Silver fluoride-AgF (Orthorhombic)	5.6%	3.7%	**×**	**×**
Calcium silver aluminum- Ca_3_Ag_12_Al_2_ (Cubic)	14.1%	**×**	**×**	**×**

SDF: Silver diamine fluoride; SNF: Stannous chloride-containing fluoride solution; DW: Deionized water; Conc: Concentration; **×**: Not available

### Elemental composition

**Fig. 3:**
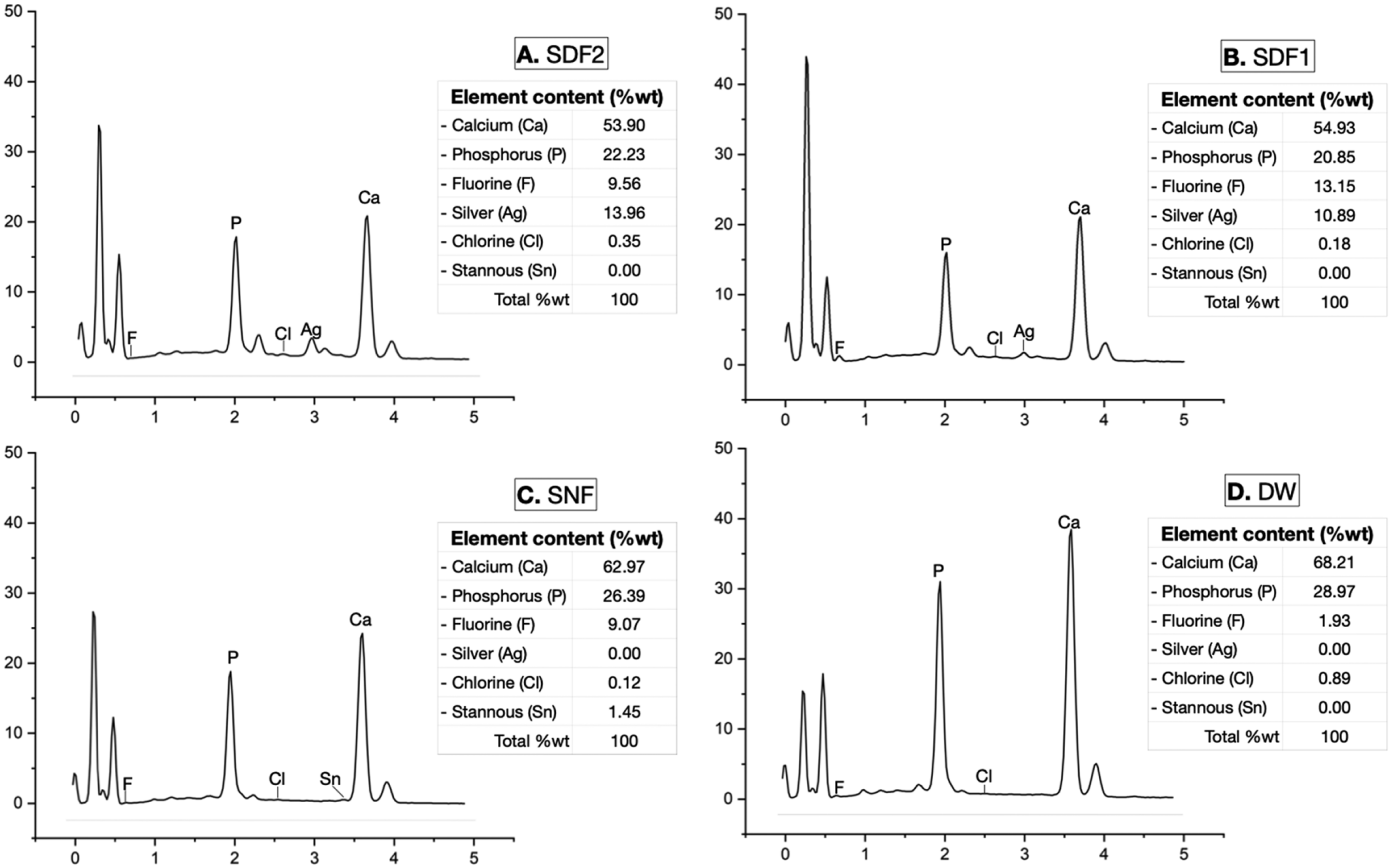
Elemental analysis of dentin in the four experimental groups (**A**: SDF2, **B**: SDF1, **C**: SNF, and **D**: DW) by Energy-dispersive X-ray spectroscopy (EDS). SDF: silver diamine fluoride; SNF: stannous chloride-containing fluoride solution; DW: deionized water

Figure 3 shows the EDS spectrograms of the precipitated layer on the dentin blocks in four experimental groups. EDS detected the peaks of calcium, phosphorus, chlorine, and fluorine elements in all groups. The EDS spectrograms showed a difference in the levels of calcium and phosphorus elements among the four experimental groups. The silver element was detected on the surfaces of the dentin blocks in Groups SDF2 and SDF1, indicating the formation of silver compounds on the surface of the dentin blocks. However, the EDS analysis detected a high peak of silver in Group SDF2. A stannous peak was detected on the surfaces of dentin blocks only in Group SNF. Moreover, the increased weight% of fluorine element in Group SNF, SDF2, and SDF1 was detected compared to Group DW.

### Surface morphology

**Fig. 4:**
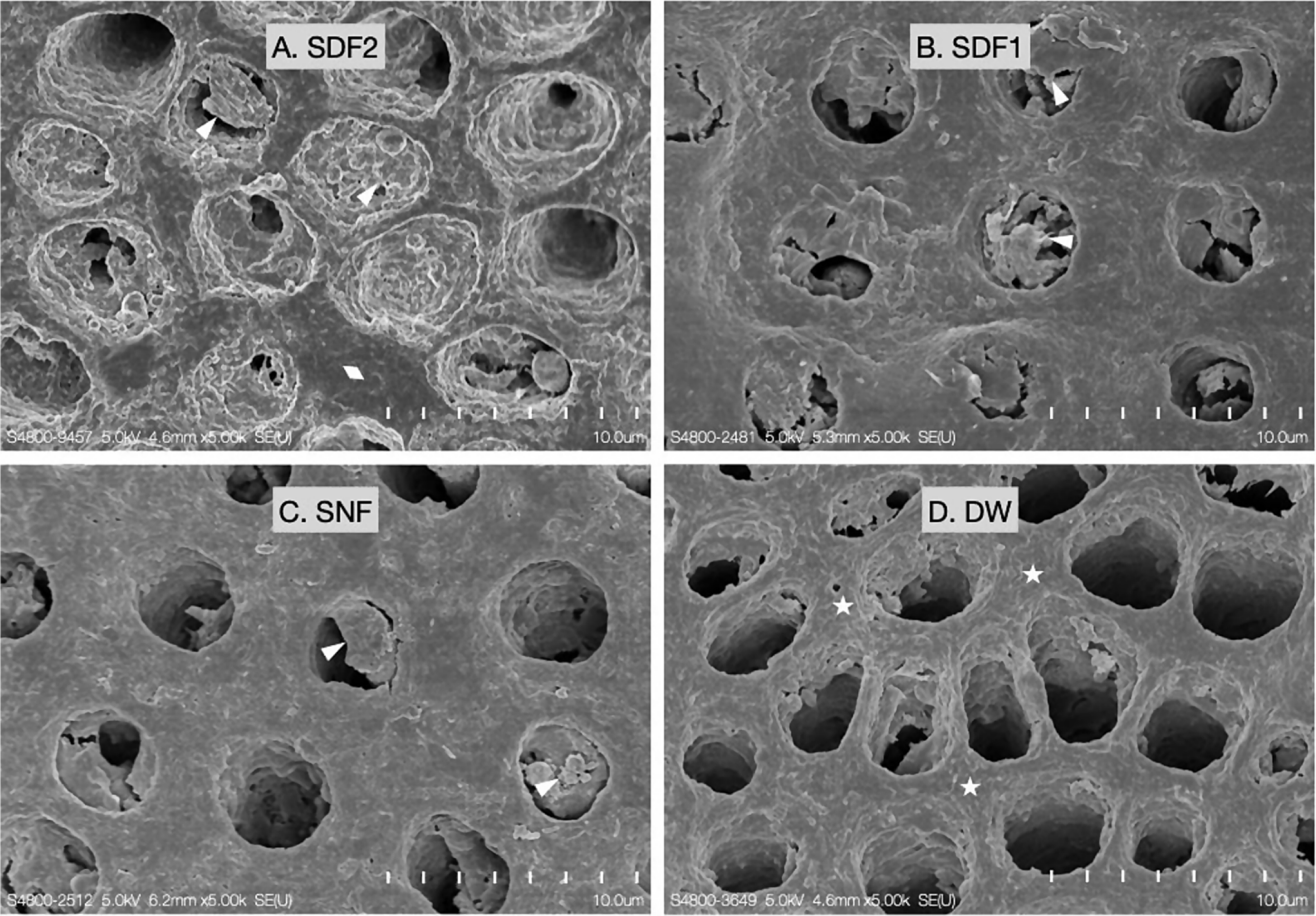
Representative scanning electron micrographs of the dentin surface morphology in the four experimental groups (**A**: SDF2, **B**: SDF1, **C**: SNF, and **D**: DW) under 5,000X magnification. SDF: silver diamine fluoride; SNF: stannous chloride-containing fluoride solution; DW: deionized water. (Δ= Occluding material; ◊ = precipitated layer; * = Rough surface at inter-tubular area)

SEM images of the dentin surface morphology of four experimental groups is shown in Fig. 4.

In the SEM image of the dentin surface in Group DW, it was observed that inter-tubular areas had relatively rough surfaces with the exposure of the reticular nanostructure of the collagen fibers. Exposed collagen fibers and rough surfaces in Group DW indicated that the surface was demineralized. The SEM images of the dentin surfaces in Groups SDF2, SDF1, and SNF had relatively smooth surfaces of inter-tubular areas. In addition, dentinal tubules in Groups SDF2, SDF1, and SNF were occluded with plugs. Meanwhile, the dentin surface of Group DW had no dentinal occlusion with opening dentinal tubules.

### Surface microhardness loss

**Fig. 5:**
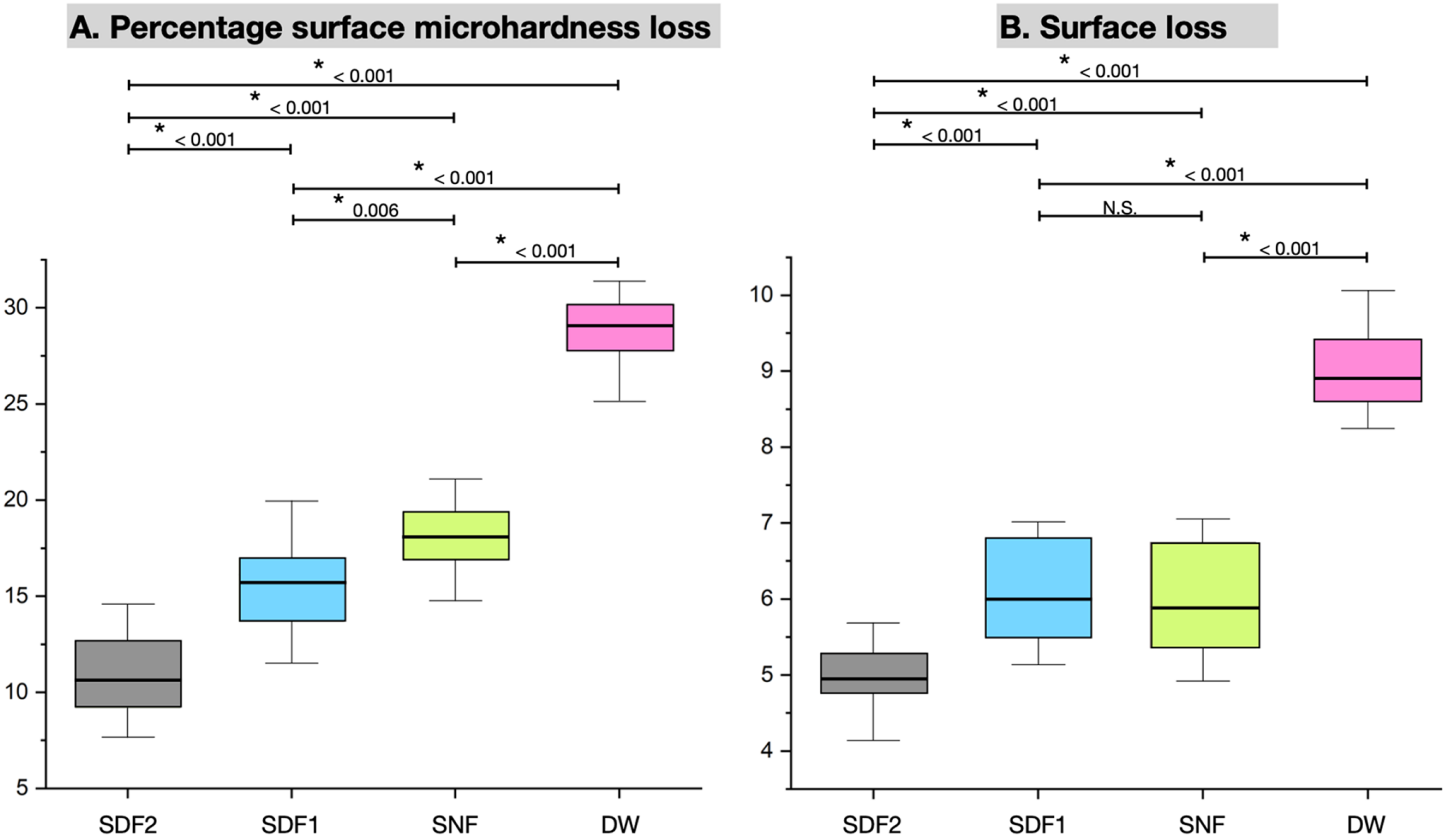
The percentage surface microhardness loss of dentin (**A**) and dentin surface loss (**B**) of the four experimental groups. The asterisk (*) indicates significant differences between groups (*p* < 0.05). SDF: silver diamine fluoride; SNF: stannous chloride-containing fluoride solution; DW: deionized water

Figure 5A shows the %SMHL of the dentin blocks in the four experimental groups. The mean dentin %SMHL (± SD) of Groups SDF2, SDF1, SNF, and DW were 10.83 ± 2.06, 15.69 ± 2.11, 17.90 ± 2.09, 28.67 ± 1.96, respectively (*p* < 0.05). The dentin blocks in Group DW had a statistically significant increase in %SMHL compared to Groups SDF2, SDF1 and SNF, (*p* < 0.001). Dentin %SMHL in Group SDF2 was statistically significantly lower than Group SDF1 (*p* < 0.001). However, dentin %SMHL in Group SDF1 was significantly lower than Group SNF (*p* = 0.006).

### Surface loss

Figure 5B shows the dentin loss in the four experimental groups. The mean dentin loss (± SD) of Groups SDF2, SDF1, SNF, and DW were 5.02 ± 0.55, 6.04 ± 0.64, 5.98 ± 0.73, 8.99 ± 0.54, respectively (*p* < 0.001). Dentin loss in Group SDF2 was significantly lower compared to Group SDF1 (*p* < 0.001). Groups SDF1 and SNF were not significantly different in dentin surface loss (*p* = 0.986). The dentin loss was highest in Group DW.

### Color change

Table 2 shows the values of color parameters (L*, a*, and b*) and the total color change (ΔE) of the dentin blocks in the four groups before surface treatment of different experimental agents and after 14-day erosive challenges. The intra-group statistical analysis of lightness L* values of dentin blocks in all groups before treatment showed significant changes compared to values after 14-day erosive challenges (*p* < 0.05). Groups SDF2 and SDF1 showed statistical difference change in a* value (*p* < 0.001). Meanwhile, Groups SNF and DW were not significantly different in a* value (*p* > 0.05). The b* values in Groups SDF1 and DW did not significantly change (*p* > 0.05). On the contrary, Groups SDF2 and SNF showed significant change (*p* < 0.05).

The mean ΔE ± SD in the dentin blocks in Groups SDF2, SDF1, SNF, and DW were 25.98 ± 3.42, 12.07 ± 3.79, 6.85 ± 3.54, 3.87 ± 3.64, respectively. The dentin blocks in Group SDF2 revealed the highest ΔE value, whereas the blocks treated with DW yielded the lowest ΔE value. Group SDF2 showed a significantly higher total color change than Group SDF1 (*p* < 0.001). However, ΔE values in Groups SNF and DW were not significantly different (*p* = 0.051).

**Table 2:** The mean (± SD) values of coloring parameters of dentin blocks in the four groups

Group	Coordinates	Before treatment	After 14 days challenges	*p* value
SDF2	L* a* b*	92.01 ± 4.47 2.56 ± 1.29 26.41 ± 4.88	67.15 ± 6.99 6.84 ± 0.66 17.60 ± 2.75	< 0.001 < 0.001 < 0.001
SDF1	L* a* b*	92.71 ± 3.61 1.59 ± 0.54 25.86 ± 5.90	78.98 ± 4.95 4.14 ± 1.00 28.93 ± 4.77	< 0.001 < 0.001 0.073
SNF	L* a* b*	90.89 ± 5.23 1.73 ± 1.30 24.05 ± 3.53	81.93 ± 4.82 2.87 ± 2.29 28.93 ± 7.06	< 0.001 0.108 0.016
DW	L* a* b*	92.61 ± 7.22 3.23 ± 3.15 24.82 ± 5.48	89.51 ± 6.51 3.54 ± 2.25 21.94 ± 4.19	0.003 0.607 0.053

The L* axis represented lightness ranging from black (0) to white (100), the a* axis represented red (+ a*) to green (-a*), and the b* axis represented yellow (+ b*) to blue (-b*). The total color change (ΔE) was calculated according to the formula of ΔE = {(L_before_*-L_after_*)^2^ + (a_before_*-a_after_*)^2^ + (b_before_*-b_after_*)^2^}^1/2^

SDF: Silver diamine fluoride; SNF: Stannous chloride-containing fluoride solution; DW: Deionized water

## Discussion

This study is the first study to examine the frequency of 38% silver diamine fluoride (SDF) application in preventing dental erosion in human dentin. Our results supported that the increased frequency of SDF applications promoted the effectiveness of SDF in preventing dental erosion on the dentin blocks. Thus, the null hypothesis was rejected. The results of this study could contribute to future studies for developing of the application protocol of 38% SDF solution for preventing dentin erosion.

The significant finding of XRD on the SDF-treated dentin blocks was silver compounds, such as silver phosphate (Ag_3_PO_4_), silver chloride (AgCl), and silver fluoride (AgF), on both dentin surfaces in Groups SDF2 and SDF1. Calcium silver aluminum (Ca_3_Ag_12_Al_2_) was found only on the dentin surfaces in Group SDF2. After SDF re-application on the dentin surfaces in Group SDF2, a large amount of fluoride and silver ions were re-supplied to the dentin surfaces. Hence, the XRD analysis exhibited an up-regulation of silver fluoride concentration level and a down-regulation of silver phosphate and silver chloride concentration levels compared to Group SDF1. In addition, this study used fresh human saliva, which contains calcium and phosphorus ions [16, 43, 44]. Therefore, all mineral ions in human saliva could exchange, react, and incorporate with SDF on the dentin surfaces [16].

The increased frequency of SDF influenced the crystallization of hydroxyapatite in the tooth surface. The XRD pattern demonstrated the increased intensity of the peak of hydroxyapatite in Group SDF2. It was confirmed that SDF could react with hydroxyapatite to provide better crystallization and these findings were consistent with previous studies [16, 45, 46]. Surprisingly, fluorapatite and calcium fluoride (CaF_2_) were not detected on the XRD peak pattern in the present study. It was inconsistent with the previous studies [16, 46, 47]. The reason could be that calcium fluoride has a high solubility and is easy to detach after acidic challenge [9, 10]. Moreover, calcium fluoride and fluorapatite can serve as a fluoride-releasing reservoir to react with other ions to form different compounds [16, 48]. Nevertheless, calcium fluoride, sodium fluoride, ammonium fluoride, and stannous chloride were formed on the blocks of Group SNF. These compounds had higher solubility than silver compounds, but SnCl_2_/AmF/NaF solution was applied daily; thus, these compounds were replenished on the blocks.

The elemental compositions on the surface of dentin blocks were assessed by EDS. The weight% of silver and fluorine elements were found on dentin surfaces in Groups SDF2 and SDF1 in EDS results. The reason could be explained as follows: the increased frequency of SDF application provided more silver and fluoride ions to re-supply on the SDF-treated dentin surfaces. Therefore, the concentration level and weight% of the silver element on dentin surfaces in Group SDF2 were higher than in Group SDF1. Meanwhile, the EDS results of the dentin surfaces in Group SNF found that stannous and fluorine elements were detected due to the agent’s ingredients.

SEM images of SDF-treated dentin surfaces in Groups SDF2 and SDF1 exhibited dentinal tubule occlusion and a precipitated layer at intertubular dentin. The finding was consistent with the reports by Mei et al. 2013 [49], Li et al. 2019 [50], and Seto et al. 2020 [51]. Silver did not change the microstructure of the treated dentin but rather filled in the space of the tubule and precipitates on the dentin surface [51]. Sparse aggregated structure of silver particles extended into dentinal tubules [49, 51]. It could extend approximately a few hundred micrometers into dentinal tubules [49, 51]. These precipitations could protect the dentin structure from erosive demineralization [10]. The SEM results of the dentin surfaces supported that the increased frequency of SDF application on the dentin surface promoted the formation of silver plugs in the dentinal tubule and the precipitated layer on the dentin surface.

Likewise, the SEM image of the SNF-treated surface in Group SNF demonstrated dentinal tubular occlusion similar to the SDF-treated surface. It was consistent with the reports by da Silva et al., 2017 [52] and Machado et al. 2020 [53]. Stannous chloride containing fluoride can precipitate onto dentin surface and dentinal tubule [10, 52]. The occluded dentinal tubule and precipitated layers can protect the dentin surface from acid attack [10, 52, 53]. However, the occluded and precipitated layer of the SNF-treated dentin surface had low solubility and was easy to remove compared to silver compounds of the SDF-treated dentin surface [10]. On the contrary, the peritubular of Group DW was extensively damaged by erosive attacks. This can be explained by the higher mineral content of peritubular dentin than intertubular dentin, which contains more than 40% [54]. Consequently, the SEM image of Group DW showed an enlarged dentinal tubule and exposed some intratubular dentin by acidic demineralization.

The surface microhardness loss of SDF-treated dentin was significantly lower compared to SNF and DW-treated dentin surfaces. The microhardness test indirectly determines mineral changes in hard dental tissue [55]. Therefore, surface microhardness loss was used to evaluate the preventive effect of the experimental agents against dental erosion. The results of the surface microhardness loss in this study could be due to the chemical reaction of SDF with the dentin surfaces, which promoted hydroxyapatite crystallization and silver compounds formation on the dentin surfaces [16, 45, 46]. In addition, after SDF application, SDF-treated dentin blocks were immersed in fresh human saliva. The silver and fluoride ions of SDF reacted with hydroxyapatite of the dentin structure and mineral ions in fresh human saliva to form some precipitated layer of the silver compound, such as silver phosphate, silver chloride, and silver fluoride, as reported in XRD results. These silver compounds had high stability and insolubility, contributing to higher acid resistance of the SDF-treated dentin surfaces [42, 45]. These findings were consistent with the previous studies [9, 48]. Meanwhile, SNF-treated dentin surfaces were found to have sodium fluoride (NaF), calcium fluoride (CaF_2_), ammonium fluoride (NH_4_F), and stannous chloride (SnCl_2_), which were highly soluble compounds [10]. These compounds have been known to increase the protection on dentin surfaces in the short term but could not resist multiple erosive challenges [9, 16].

When comparing the surface microhardness loss value between two groups of SDF-treated dentin surfaces. The results showed that Group SDF2 was statistically significantly lower than Group SDF1, indicating that the increased frequency of SDF application provided more protection against mineral loss [9, 10]. The second time of SDF application might enhance surface hardness by re-supplying silver and fluoride ions, which offered another chance of remineralization by preserving phosphate and calcium ions on eroded dentin surfaces [56–58]. Moreover, SDF facilitated the formation of covalent bonds between ions in SDF and phosphate groups on exposed collagen fibrils and protein networks [22, 58]. Thus, SDF contributed to increased calcium and phosphate in eroded dentin [58]. Therefore, the increased frequency of SDF application increased the mineral content and density on eroded dentin surfaces [25] and impeded mineral dissolution of the SDF-treated dentin surfaces.

The results of dentin surface loss value in this study showed that both groups of SDF had significantly lower dentin surface loss than Groups SNF and DW. The non-contact profilometer is a gold standard for measuring the depth of irreversible dentin surface loss and was used to compare the anti-erosive properties of experimental agents [34, 59]. The reduced surface loss in SDF-treated dentin could be explained as follows: SDF can form a more stable and acid-resistant precipitated layer due to its higher silver compound composition [9, 10, 60]. The precipitated layer of SDF could be a physical barrier on the block’s surface, thereby inhibiting direct contact and delaying the diffusion between erosive acids and the block’s surface [9, 10]. Additionally, SDF is a clear solution with high flowability and liquidity [36, 45]. It provides 2–3 times more fluoride than other types of fluoride on the applied surface [61]. The silver and fluoride ions from SDF can penetrate 200–300 μm into the dentin [25] through dentinal tubules and micropores in the dentin block [21, 54]. Then, SDF can react with calcium and phosphorus ions along the dentin tubule and dentin surface [21, 54]. It also exchanged mineral ions during immersion in fresh human saliva [16, 22, 58]. In addition, SDF can incorporate and modify hydroxyapatite to form silver compounds, such as silver phosphate, silver chloride, and silver fluoride [9, 46]. These reactions increased the resistance of SDF-treated dentin surfaces to demineralization. In addition, SDF has been known as a collagenase and matrix metalloproteinase inhibitor [49]. When interfacing with a low pH, SDF preserves the collagen matrix on the dentin surfaces [21]. Therefore, SDF was effective in reducing dentin surface loss due to erosive demineralization.

The increased frequency of SDF application in Group SDF2 reduced dentin surface loss compared to a single application of SDF in Group SDF1. It could be explained that the alkaline pH of SDF (~ 10) at the second application of SDF could promote apatite nucleation on the eroded dentin [22, 58]. Besides, the mineral crystals of eroded dentin served as nucleation sites for the crystallization of the remineralizing process [22, 58, 62]. The increased application frequency of SDF provided more fluoride and silver ions from SDF [16, 22, 58]. Hence, these ions reacted with calcium and phosphate ions from human saliva to deposit on the nucleation sites of eroded dentin [16, 22, 58]. The collagen fibrils and protein networks were exposed to eroded dentin [22]. The increased application frequency of SDF also enhanced the inhibition of matrix metalloproteinases activity of SDF [22, 30]. The re-supply of silver ions from the second time of SDF application might further react with the exposed proteins within the eroded dentin structure [30, 58] and form silver-protein complexes that might protect dentin collagen from further demineralization [30].

The results of color change (ΔE) in this study showed that the dentin blocks treated with SDF had a profound color change compared to other experimental treatments. The L* values of the dentin surfaces in all groups showed a statistical difference from baseline. It could be explained by aging in relation to the baseline reading, black metallic silver compounds from SDF application in Groups SDF2 and SDF1 and stannous compounds from SNF application in Group SNF influenced the dramatic L* value change [40–42, 63, 64]. Changes in a* and b* values in Groups SDF2, SDF1, and SNF represented varied significantly, which can be explained by the fact that these experimental agents may have photosensitivity effects [40–42]. Moreover, spectrophotometer measures the visible spectrum in the L* a* b* color system. The results of L*, a*, and b* values from the spectrophotometer might be affected by the different positions of the left and right shift of the spectrophotometer and the different areas on the block surface before the experiment treatment and after the 14-day erosive challenge [40–42].

Additionally, this study used the mid-coronal part of tooth to obtain dentin blocks, which exposed the dentinal tubule and intertubular dentin. Each dentin block was polished to obtain a flat surface. Hence, applying SDF on the polished dentin surface may accelerate the black staining at the first application of SDF. Although stannous containing fluoride solution has been reported to cause extrinsic tooth staining due to incomplete stabilization of stannous ions on the surface [63, 64], the ΔE of the dentin surfaces in Group SNF was not a statistically significant difference from Group DW in this study. However, the ΔE value of dentin surfaces in Group SDF2 was statistically higher than ΔE in Group SDF1. It could be explained that the polished dentin surfaces were roughened after a 7-day erosive challenge. The reapplication of SDF on Day 8 of the experiment provided more silver ions on the SDF-treated roughened surfaces. The silver ions could deposit and play a significant role in causing an intense black stain on the roughened dentin surface [9, 41]. It should be noted that black staining resulting from SDF application is an unavoidable side effect and could be a major concern for patients [23, 41].

The present study has limitations, this study was conducted in a relatively short period of 14 days. Therefore, the frequencies of SDF applications were set as a single application and two weekly applications. The effect of other frequencies of SDF applications on dental erosion prevention with a longer experimental span could be investigated. In addition, there is no consensus on how to use SDF for anti-erosive purposes. Thus, the protocol of this study followed the dental caries management protocol, which recommends applying SDF for one minute on one surface [25]. However, it might take longer for the chairside time to apply on multiple tooth surfaces for anti-erosive purposes in clinical situations. Moreover, this study simulated dental erosion on dentin blocks without mechanical forces. The effect of SDF on preventing erosive tooth wear should be investigated in future studies [2].

## Conclusion

38% SDF with two weekly applications provided better preventive effects against dentin erosion compared to a single application, but it might discolor dentin.

## Data Availability

Data is provided within the manuscript or supplementary information files.
